# Editorial: society journals matter—supporting science through renewed commitment

**DOI:** 10.1093/femsml/uqaf032

**Published:** 2025-11-19

**Authors:** Paul B Rainey, Puri López-García, Zeynep Ceren Karahan, Paul Williams, Stipan Jonjić, Kenneth N Timmis

**Affiliations:** Department of Microbial Population Biology, Max Planck Institute for Evolutionary Biology, Plön 24306, Germany & Laboratory of Biophysics and Evolution, CBI, ESPCI Paris, Université PSL, CNRS, Paris 75005, France; Ecologie Société Evolution, CNRS, Université Paris-Saclay, AgroParisTech, Gif-sur-Yvette 91190, France; Department of Medical Microbiology and Ibn-i Sina Hospital Central Microbiology Laboratory, Ankara University School of Medicine, Ankara 06230, Turkey; Biodiscovery Institute and School of Life Sciences, University of Nottingham, Nottingham NG72RD, United Kingdom; Center for Proteomics, Faculty of Medicine, University of Rijeka, Rijeka 51000, Croatia; Institute of Microbiology, Technical University Braunschweig, Braunschweig 38106, Germany

Scientific publishing is in flux. Commercial publishers have expanded their influence, driven by profit margins and citation metrics. At the same time, a growing ecosystem of predatory journals has emerged, exploiting publish-or-perish pressures by offering speed and a veneer of legitimacy without meaningful editorial standards.

In response to these challenges, the European Academy of Microbiology (EAM) established a Task Force on predatory publishing. One of its conclusions is that predatory outlets cannot be understood in isolation: they exist because there is demand. That demand is shaped by career and evaluation pressures, frustrations with conventional publishing, the behaviour of a minority of opportunistic authors, and the incentives facing learned societies. Several of these themes are treated in companion op-eds (Timmis et al. 2025, de Lorenzo *et al*. 2025, Rainey et al. 2025); here we focus on learned societies and their journals.

## Why society journals matter

Journals owned and run by scientific societies remain among the few venues committed to serving the research community. Revenues from society journals fund conferences, training programs, policy engagement, and early-career support. Editors are scientists and disciplinary experts, guided by scholarly judgement rather than market imperatives. The integrity of review and decision-making is aligned with the long-term interests of the discipline and not with short-term metrics.

Despite their importance, society journals now publish only a fraction of the work produced in their fields. Schloss and colleagues (2017 et al.) showed that for microbiology, the number of papers appearing in society journals has plateaued, while publication in commercial mega-journals has surged. This imbalance matters, because every paper diverted to for-profit outlets is one less supporting community activities and values.

## Navigating the impact factor game

A paper that addresses an open question, is methodologically sound, carefully reasoned, and advances understanding—even incrementally—should be deemed worthy. However across the publishing landscape, pressures to compete in the ‘impact factor game’ risk pulling even society journals away from this principle (Trueblood et al. 2025). Papers may be judged not on their intrinsic quality, but on their perceived ability to deliver ‘excitement’ or citations. While understandable in a prestige-driven environment, this can undervalue the steady, cumulative contributions on which science depends.

Many societies have recognized this tension and created companion journals to accommodate rigorous but less ‘headline-grabbing’ work: the American Society for Microbiology (ASM) with *mSphere*, the Royal Society with *Open Science*, and the National Academy of Sciences with *PNAS Nexus*. These step-down journals are valuable because they provide legitimate outlets and help keep sound work within the society ecosystem. However, they are also symptomatic of a deeper problem: flagship titles, under pressure to maximize impact factors, sometimes push worthy work—labelled "unexciting"—into secondary tiers.

The effects of such selectivity are not borne equally. Science across the globe is not evenly funded, and many laboratories in less well-resourced countries cannot routinely access the latest technologies. Nonetheless, work from such settings can still be rigorous, careful, and insightful. When novelty or technological sophistication is taken as a proxy for worth, this kind of science is easily sidelined. Too often, it ends up in predatory outlets—not because of poor quality, but because the system offers few alternatives. Society journals, committed to serving the whole community, could do well to recognize this problem and develop solutions that keep such contributions within the legitimate scientific record.

Some society journals are already charting a different path. For example, ASM’s *Microbiology Spectrum* explicitly rejects subjective novelty criteria, promising instead to publish research that is technically sound and useful to the community. Such models illustrate that societies can resist the pull of prestige-driven publishing while upholding quality and inclusivity.

## Balancing sustainability with service

None of this is to argue for lowering standards. The opposite is true. Upholding rigour, clarity, transparency, and reproducibility is the baseline. But it is important to resist conflating scientific worth with newsworthiness and overhype. As historians of science have argued, reliability is often a better measure of value than novelty (Oreskes 2019). And as numerous authors have noted, the obsession with impact factors continues to distort incentives and discourage publication of sound, worthy research (Casadevall and Fang 2014, Dougherty and Horne 2022, Rainey et al. 2025, Trueblood et al. 2025).

Alongside editorial choices, society journals face deeper structural issues. Many were founded to serve members and the discipline: prioritizing member submissions, charging modest fees, and reinvesting proceeds into the community. But as commercial publishers pursued ever-larger profits, some societies followed suit. In fact in some instances, societies sold their journals to publishing houses in return for a fraction of profits, and thus relinquished autonomy. Journal revenues grew—not always to support science, but to expand bureaucracies. In the name of becoming more ‘professional’, administrative overheads ballooned, with a growing share of revenues now funding salaries, pensions, and institutional growth. That might be defensible if member services expanded accordingly. In many cases, they haven’t.

As the shift to open access disrupts traditional revenue streams, some societies are responding by cutting core academic activities, raising article processing charges (APCs) and tightening selectivity in pursuit of visibility. Such strategies may provide short-term stability, but they also blur the line between serving members and competing in a prestige economy. At a time when commercial publishers extract monopolistic profits (Buranyi 2017, Haustein et al. 2024), society journals risk drifting from their founding mission if they chase the same measures of success.

At the same time, there are constructive innovations. Read-and-publish agreements, such as those introduced by the Microbiology Society in the UK, help reduce costs for authors while sustaining society journals. Such approaches demonstrate that societies can adapt in ways that protect both financial sustainability and accessibility for their members.

## Reaffirming the community role of society journals

What societies need is not simply to preserve revenue streams, but to ask how best to serve their members and fields within available means. That means affordable publishing, prioritising support for under-resourced members, and valuing rigorous work regardless of perceived impact (Field 2022). If society journals allow impact-factor considerations to dictate selectivity, they undermine their credibility as community journals and drive authors towards less scrupulous alternatives.

Addressing inequities in global science is part of this responsibility. Ensuring that sound work from underfunded contexts has a fair hearing, whether through editorial philosophy, affordable APCs, or appropriate journal scope, is not just an act of inclusion, but a safeguard against predatory publishing.

Sustaining society journals; however, is not only the responsibility of the societies themselves. As authors, reviewers, and members, we also have a role. Too many sound papers, unable to reach the selective ‘prestige journals’, now flow into for-profit outlets where revenues do not return to the community. These papers could and should be published in society journals. Choosing our professional home-journals strengthens the societies that train early-career scientists, organize meetings, and advocate for the discipline.

Innovations such as read-and-publish agreements show that change is possible, but they will only succeed if researchers commit to supporting their own journals. If we fail to act, the gap will be filled not only by commercial houses but by predatory publishers. If we succeed, society journals can continue to serve as credible, trusted venues that keep science both rigorous and inclusive.

Now is the time for society journals to lead by reaffirming the principles on which they were founded. That also means mentoring young scientists and embodying the practices we advocate, so that values of rigour and service are sustained across generations. And it is time for us, as scientists, to support our society journals by submitting, reviewing, and ensuring that our professional home journals remain central to the life of our disciplines.

## References

[bib1] Buranyi S. Is the staggeringly profitable business of scientific publishing bad for science?. *The Guardian*, 27 June 2017. 2017. https://www.theguardian.com/science/2017/jun/27/profitable-business-scientific-publishing-bad-for-science (29 October 2025, date last accessed).

[bib2] Casadevall A, Fang FC. Causes for the persistence of impact factor mania. mBio. 2014;5:e00064–14. 10.1128/mbio.00064-14. 24643863 PMC3967521

[bib3] de Lorenzo V, Rainey PB, Willliams P et al. Storm over science: predatory practices and the fight for research reliability. μLife. 2025;6:uqaf029. 10.1093/femsml. PMC12539563 41127409

[bib4] Dougherty MR, Horne Z. Citation counts and journal impact factors do not capture some indicators of research quality in the behavioural and brain sciences. R Soc Open Sci. 2022;9:220334. 10.1098/rsos.220334. 35991336 PMC9382220

[bib5] Field SM. Charting the Constellation of Science Reform. PhD Thesis, Groningen, Netherlands: University of Groningen, 2022.

[bib6] Haustein S, Schares E, Alperin JP et al. Estimating global article processing charges paid to six publishers for open access between 2019 and 2023. 2024. 10.48550/arXiv.2407.16551.

[bib7] Oreskes N. Why Trust Science?. Princeton, USA: Princeton University Press, 2019.

[bib11_541_045425] Rainey PB, López-García P, de Lorenzo V et al. Pursuit of knowledge: A charter for academic renewal. Zenodo. 2025. 10.5281/zenodo.17488783.

[bib9] Schloss PD, Johnston M, Casadevall A. Support science by publishing in scientific society journals. mBio. 2017;8:e01633–17. 28951482 10.1128/mBio.01633-17 PMC5615203

[bib10] Timmis K, Williams P, Karahan ZC et al. Journals operating predatory practices are systematically eroding the science ethos: a gate and code strategy to minimise their operating space and restore research best practice. Microb Biotechnol. 2025;18:e70180(8). 10.1111/1751-7915.70180. 40536143 PMC12177670

[bib11] Trueblood JS, Allison DB, Field SM et al. The misalignment of incentives in academic publishing and implications for journal reform. Proc Natl Acad Sci USA. 2025;122:e2401231121. 10.1073/pnas.2401231121. 39869806 PMC11804702

